# Temporal changes in electrocardiographic frontal QRS-T angle and survival in patients with heart failure

**DOI:** 10.1371/journal.pone.0194520

**Published:** 2018-03-26

**Authors:** Israel Gotsman, Ayelet Shauer, Yair Elizur, Donna R. Zwas, Chaim Lotan, Andre Keren

**Affiliations:** 1 Heart Failure Center, Heart Institute, Hadassah University Hospital, Jerusalem, Israel; 2 Heart Failure Clinic, Clalit Health Services, Jerusalem, Israel; Kurume University School of Medicine, JAPAN

## Abstract

**Background:**

Heart failure (HF) is associated with considerable mortality. The electrocardiographic frontal QRS-T angle is a simple parameter to measure, reflects changes in the direction of the repolarization sequence and predicts outcome in patients with HF. Data regarding temporal changes in the frontal QRS-T angle in patients with HF and its impact on outcome is limited.

**Aim:**

To evaluate temporal changes in the frontal QRS-T angle and its effect on survival in patients with HF.

**Methods:**

Baseline and follow-up QRS-T angle were calculated from the frontal QRS and T axis of the 12-lead surface electrocardiogram. Patients were followed for survival.

**Results:**

2,929 HF patients were evaluated. Median interval between baseline ECG and follow-up ECG was 895 days, median follow-up time was 1526 days. Overall, the QRS-T angle tended to be stable, with minor changes in the angle over time. The median QRS-T angle change was +3° (IQR -19° to +30°). Overall survival during follow-up was 60%. Cox regression analysis after adjustment for significant predictors demonstrated that the QRS-T angle was an incremental predictor of increased mortality. A widening of the QRS-T angle during follow-up was independently associated with an increase in mortality, evident with an increase of the QRS-T angle difference above 0° (P<0.0001 for the adjusted model).

**Conclusion:**

QRS-T angle is relatively stable in patients with HF and is a powerful predictor of outcome. Widening of the QRS-T angle has predictive value and is an ominous sign.

## Introduction

Heart failure (HF) is an epidemic with a high rate of hospitalizations and death [1]. The electrocardiogram (ECG) can provide important information on structural and electrophysiological abnormalities in patients with HF. The spatial QRS-T angle, the angle between the directions of ventricular depolarization and repolarization, represents abnormal cardiac structure and electrical heterogeneities resulting in changes of the repolarization direction. Due to this, it is a strong marker of electrical instability and susceptibility to ventricular arrhythmias. The spatial QRS-T angle has been shown to predict cardiovascular death in the general population [2, 3] and is the strongest electrocardiographic predictor of cardiovascular death in women [4]. The frontal QRS-T angle, a simplified and more obtainable parameter, conveys comparable prognostic information [5, 6]. The frontal QRS-T angle predicts arrhythmic events in patients with left ventricular dysfunction [7, 8] and is a predictor of outcome in patients with chronic HF [9] as well as in patients with diastolic HF [10]. Data regarding temporal changes in the frontal QRS-T angle in patients with HF and its impact on outcome is limited. The purpose of the present study was to evaluate changes in the frontal QRS-T angle and its effect on survival in patients with HF.

## Methods

The data was obtained from a central computerized database of the largest health maintenance organization (HMO), Clalit Health Services in Jerusalem, Israel, as described previously [9]. The database records digitally all data on the members of the HMO and includes demographics, comprehensive clinical data, diagnoses, and all laboratory data undertaken in a single centralized laboratory of the HMO. We retrieved electronically from the database all members with a diagnosis of HF as coded by the database using the International Classification of Diseases, Ninth Revision (ICD-9) code 428. 6,946 patients had a diagnosis of HF. Validation of the diagnosis of HF was performed on a randomly computer-generated 5% of the diagnosed HF patients (N = 338) as previously described[11]. In this group, 99% fulfilled the European Society of Cardiology (ESC) criteria for the diagnosis of HF[12]. We retrieved the first ECG performed in these patients within 6 months closest to July 2008, the time the database was established. We also retrieved the last ECG available. Patients were followed until September 2012, end of the clinical follow-up time. 2,929 (42%) of the patients had a follow-up ECG for analysis. These individuals compromised the cohort of this study. Echocardiography data pertaining to left ventricular (LV) systolic function was available digitally in 45% of the cohort (N = 1,306) and a separate analysis was performed on this population. Data on mortality was retrieved from the National Census Bureau. The Institutional Committee for Human Studies of the HMO—Clalit Health Services, approved the study protocol.

ECGs were acquired using the MAC 5500 ECG Diagnosis System (Marquette Electronics, Milwaukee, Wisconsin) and standard 12-lead ECGs were recorded using standardized procedures. ECGs were analyzed using the GE Marquette 12-SL ECG Analysis Program (Marquette 12SL ECG Physician Guide). All parameters analyzed were derived from this program. Frontal plane QRS-T angle was defined as the absolute value of the difference between the frontal plane QRS axis and T axis and was adjusted to an acute angle by (360°—angle) for an angle larger than 180°.

Echocardiography data were performed according to standard recommendations of the American Society of Echocardiography and the European Association of Cardiovascular Imaging [13] and were evaluated and verified by qualified personnel. For descriptive purposes LV systolic function was divided into categories based on the LV ejection fraction (LVEF) (≥50%, 40–49%, 30–39% and <30%).

SPSS version 17.0 for Windows (SPSS Inc., Chicago, Illinois, USA) and R Statistical Software version 3.0.1 for Windows (R Development Core Team) was used for the analyses. Log_10_ was used for logarithmic transformations. Follow-up time was calculated using Kaplan-Meier estimate of potential follow-up [14]. Kaplan-Meier curves, with the log-rank test, were used to compare survival according to QRS-T angle categories. QRS-T angle categories were based on the baseline QRS-T angle tertiles of the cohort stratified according to gender. Multivariable Cox proportional hazards regression analysis was used to evaluate independent variables that determined survival. Parameters included in the multivariable Cox regression analysis incorporated all significant clinical and laboratory parameters with the addition of drug therapy in separate models. Parameters were entered into the model if considered relevant clinically or significant on univariable analysis. Proportionality assumptions of the Cox regression models were evaluated by log–log survival curves and with the use of Schoenfeld residuals. An evaluation of the existence of confounding or interactive effects was made between variables and their possible colinearity. Additional sensitivity analysis evaluated outcome with the exclusion of LBBB or a ventricular-paced rhythm to eliminate possible confounding of clinical outcome due to the presence of these features. Sensitivity analysis of changes in the QRS-T angle excluded patients with LBBB or a ventricular-paced rhythm in baseline ECG or follow-up ECG. Changes in the QRS-T angle was analyzed as a continuous variable using restricted cubic splines multivariable Cox regression analysis with knots at the 5^th^, 50^th^ and 95^th^ percentiles of the QRS-T angle change distribution. Kruskal Wallis test and linear regression analysis were used to evaluate the association of the QRS-T angle and left ventricular systolic function. A two-sided p-value of < 0.05 was considered statistically significant.

## Results

### Temporal changes in the QRS-T angle

**Fig 1:**
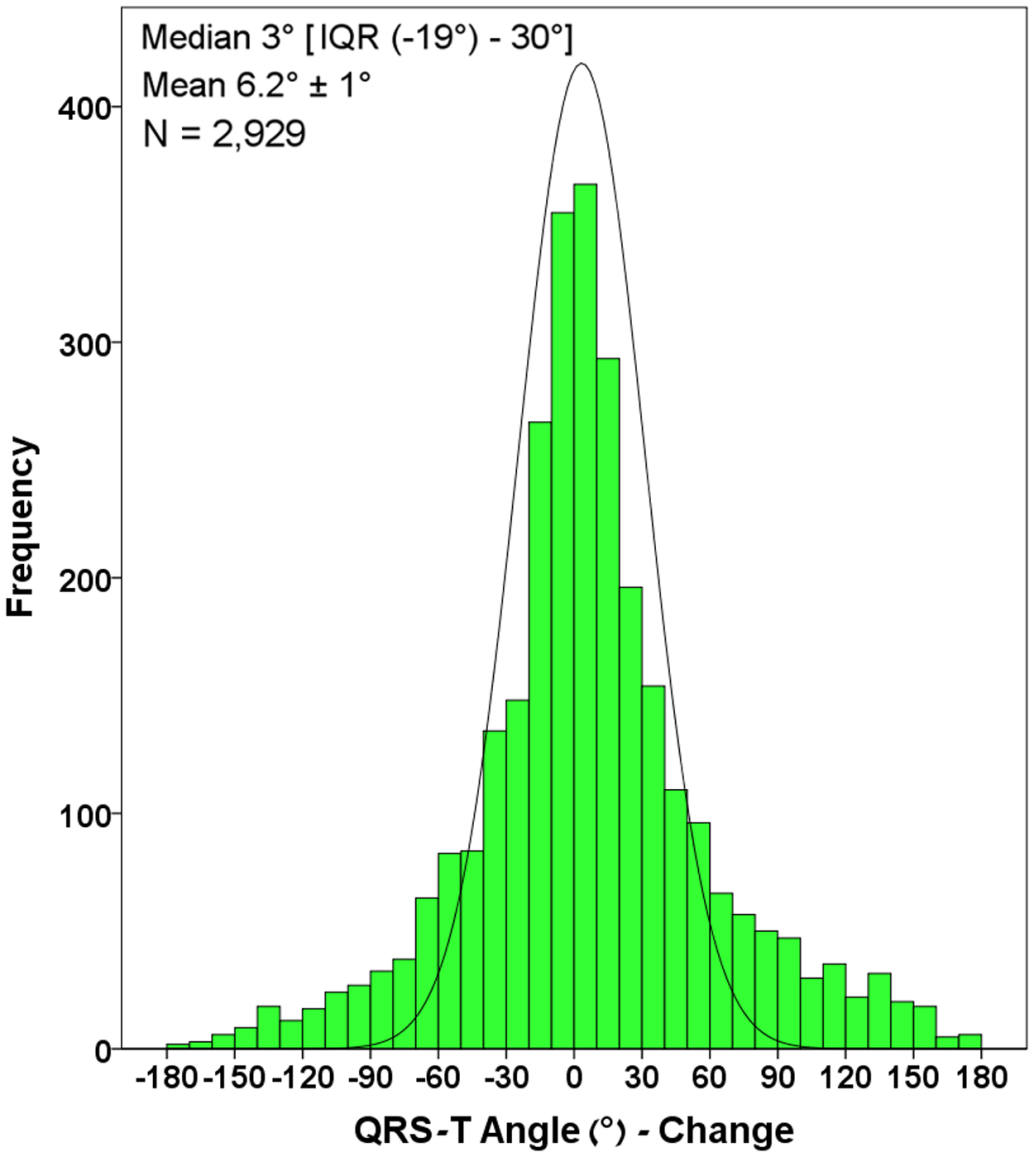
Changes in the QRS-T angle. Histogram of the QRS-T angle difference in degrees between baseline and follow-up ECG. The continuous line denotes the normal distribution curve.

The cohort of the study included 2,929 patients. Data regarding the characteristics of the patients are presented in Table 1. The median time interval between baseline ECG and follow-up ECG was 895 days (interquartile range (IQR) 316–1312 days). The median baseline QRS-T Angle was 77° (IQR 33°-135°). Median follow-up QRS-T Angle was 88° (IQR 35°-146°). Differences in the QRS-T angle during follow-up are presented in Fig 1. The median QRS-T angle change was +3° (IQR -19° to +30°). Overall, the QRS-T angle tended to be stable, with minor changes in the angle. However, 25% of the patients had an increase in the QRS-T angle of more than 30 degrees.

**Table 1:** Demographics and clinical characteristics of the patients.

Age (years)	74±13	Furosemide	2052 (70)
Gender (Men)	1551 (53)	Thiazide	738 (25)
Diabetes Mellitus	1517 (52)	Digoxin	340 (12)
Hypertension	2411 (82)	Amiodorone	507 (17)
Hyperlipidemia	2506 (86)	Aspirin	1999 (68)
Ischemic Heart Disease	2239 (76)		
Atrial Fibrillation	847 (29)	Heart rate (beats per minute)	73 (63–87)
Body Mass Index (kg/m2)	29 (28–31)	PR interval (ms)	152 (114–178)
Systolic BP (mmHg)	127 (115–139)	QRS interval (ms)	96 (84–122)
Diastolic BP (mmHg)	70 (65–79)	corrected QT interval (ms)	455 (431–483)
Pulse (beats per minute)	72 (64–80)	P axis (°)	52 (34–66)
Creatinine (mg/dL)	1.0 (0.8–1.4)	QRS axis (°)	0 ((-32)-38)
eGFR (mL/min per 1.73m^2^)	60 (42–81)	T axis (°)	62 (25–109)
Urea (mg/dL)	50 (37–73)	Baseline QRS-T Angle (°)	77 (33–135)
Hemoglobin (g/dL)	13±2	Follow-up QRS-T Angle (°)	88 (35–146)
Sodium (mEq/L)	140±3	QRS-T Angle change (°)	3 ((-19)-30)
Left ventricular ejection fraction (<50%)	736 (56)	Pacemaker	267 (9)
ACE-inhibitor / ARB	2303 (79)	Left bundle branch block	198 (7)
Beta blockers	2095 (72)	Left ventricule hypertrophy	503 (17)
Spironolactone	975 (33)	Ventricular ectopic complexes	349 (12)

Data is presented as mean ± standard deviation or median (inter-quartile range) for continuous variables and counts (percentages) for categorical variables.

### Clinical outcome

**Fig 2:**
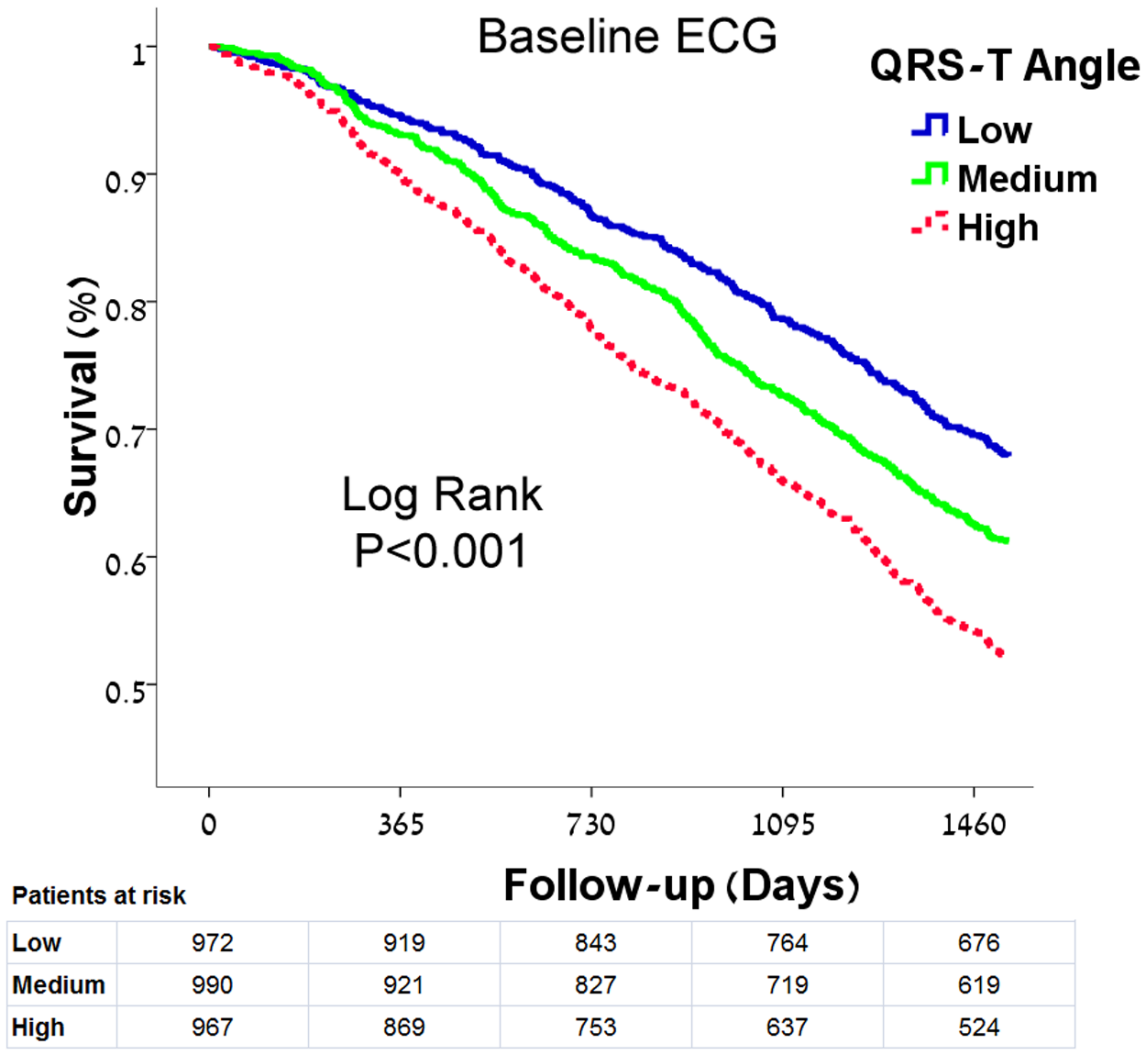
Kaplan Meier survival analysis according to baseline QRS-T angle category stratified by gender. The tertile QRS-T angle percentiles were 40° and 103° in women and 51° and 125° in men. The estimated cumulative survival rate at the median follow-up time was reduced with increasing baseline QRS-T angle category; 67.9±1.5% vs. 61.2±1.5% vs. 52.1±1.6%, P<0.001.

The median follow-up time was 1526 days. The overall mortality rate during this period was 39.6% (1,159/2,929). As there are differences in the QRS-T angle between genders we classified patients based on tertiles of the QRS-T angle in our cohort of HF patients stratified by gender. The tertile QRS-T angle percentiles were 40° and 103° in women and 51° and 125° in men. We evaluated the predictive value of this parameter stratified according to these percentiles. Increasing baseline QRS-T angle category was associated with an increased mortality rate. The estimated cumulative survival rate at the median follow-up time was reduced with increasing QRS-T angle category (Fig 2).

### QRS-T angle: Independent predictor of outcome

Multivariable Cox regression analysis after adjustment for significant predictors (see Table 2 for the predictors included) demonstrated that baseline QRS-T angle was an independent incremental predictor of increased mortality (Table 2). Analysis, after the exclusion of patients with LBBB or a ventricular-paced rhythm (N = 2560), 87% of the cohort, demonstrated that the QRS-T angle was a significant predictor of increased mortality comparing the highest to the lowest tertile (Table 3). Inclusion of HF medications did not change significantly the result and baseline QRS-T angle remained a significant incremental predictor of increased mortality (Table 3). A sensitivity analysis including only patients with left ventricular systolic function available (N = 1,306), 45% of the cohort, demonstrated a very similar result; baseline QRS-T angle was an independent predictor of survival (Table 3).

**Table 2:** Predictors of mortality by Cox regression analysis.

	Univariable		Multivariable	
	Hazard Ratio (95% CI)	P Value	Hazard Ratio (95% CI)	P Value
Age (years)	1.04 (1.04–1.05)	<0.001	1.03 (1.03–1.04)	<0.001
Gender (Male)	0.94 (0.84–1.06)	0.32	1.22 (1.06–1.40)	0.005
Diabetes Mellitus	1.17 (1.04–1.31)	0.009	1.20 (1.04–1.38)	0.01
Hyperlipidemia	0.71 (0.61–0.83)	<0.001	0.74 (0.62–0.89)	0.001
Hypertension	1.51 (1.27–1.79)	<0.001	1.20 (0.99–1.47)	0.07
Ischemic Heart Disease	1.03 (0.90–1.18)	0.70	0.89 (0.75–1.05)	0.16
Atrial Fibrillation	1.04 (0.91–1.18)	0.59	0.94 (0.81–1.08)	0.37
Body Mass Index* (kg/m^2^)	0.06 (0.03–0.13)	<0.001	0.09 (0.04–0.21)	<0.001
Pulse* (beats per minute)	3.10 (1.64–5.86)	<0.001	5.50 (2.77–10.93)	<0.001
Urea (mg/dL)*	5.62 (4.35–7.26)	<0.001	4.46 (2.78–7.17)	<0.001
eGFR** (mL/min per 1.73m^2^)	0.87 (0.85–0.90)	<0.001	1.05 (1.00–1.10)	0.07
Hemoglobin (g/dL)	0.82 (0.79–0.85)	<0.001	0.89 (0.86–0.93)	<0.001
Sodium (mEq/L)	0.94 (0.92–0.96)	<0.001	0.97 (0.95–0.99)	<0.001
QRS-T Angle		<0.001		<0.001
QRS-T Angle—Low	1.0 (Reference)		1.0 (Reference)	
QRS-T Angle—Medium	1.28 (1.10–1.48)	0.001	1.19 (1.01–1.40)	0.04
QRS-T Angle—High	1.70 (1.47–1.96)	<0.001	1.43 (1.22–1.69)	<0.001

Data is presented as hazard ratio (95% confidence interval), P value.

* Log-transformed

** Square root-transformed

**Table 3:** Hazard ratio for clinical outcome according to QRS-T angle levels by Cox regression analysis.

	QRS-T angle Category			P-value
	Low	Intermediate	High	
Univariable	1.0 (Reference)	1.28 (1.10–1.48), 0.001	1.70 (1.47–1.96), <0.001	<0.001
Multivariable	1.0 (Reference)	1.19 (1.01–1.40), 0.04	1.43 (1.22–1.69), <0.001	<0.001
Multivariable and Drugs	1.0 (Reference)	1.19 (1.01–1.40), 0.04	1.42 (1.21–1.68), <0.001	<0.001
Analysis of cohort after exclusion of patients with LBBB or paced ventricle				
Univariable	1.0 (Reference)	1.28 (1.10–1.50), 0.002	1.66 (1.42–1.95), <0.001	<0.001
Multivariable	1.0 (Reference)	1.15 (0.97–1.36), 0.11	1.39 (1.17–1.66), <0.001	0.001
Multivariable and Drugs	1.0 (Reference)	1.16 (0.98–1.38), 0.09	1.42 (1.18–1.71), <0.001	<0.001
Sub-analysis of patients with available echocardiographic data regarding left ventricular systolic function				
Univariable	1.0 (Reference)	1.36 (1.10–1.68), 0.005	1.60 (1.30–1.96), <0.001	<0.001
Multivariable	1.0 (Reference)	1.20 (0.94–1.53), 0.13	1.36 (1.08–1.71), 0.009	0.03
Multivariable and Drugs	1.0 (Reference)	1.19 (0.93–1.51), 0.16	1.33 (1.06–1.68), 0.02	0.05

Data is presented as hazard ratio (95% confidence interval), P value.

Parameters that were included in the multivariable analysis were age, gender, ischemic heart disease, diabetes, hyperlipdemia, hypertension, atrial fibrillation, log-transformed body mass index, log-transformed pulse, log-transformed serum urea levels, square root-transformed estimated glomerular filtration rate, hemoglobin, serum sodium.

Parameters that were included in the multivariable and drugs analysis included the above parameters and the drug treatment with angiotensin-converting enzyme inhibitor/angiotensin receptor blocker, beta blocker, furosemide, spironolactone, thiazide and digoxin.

### Widening of the QRS-T angle: Independent predictor of a worse outcome

**Fig 3:**
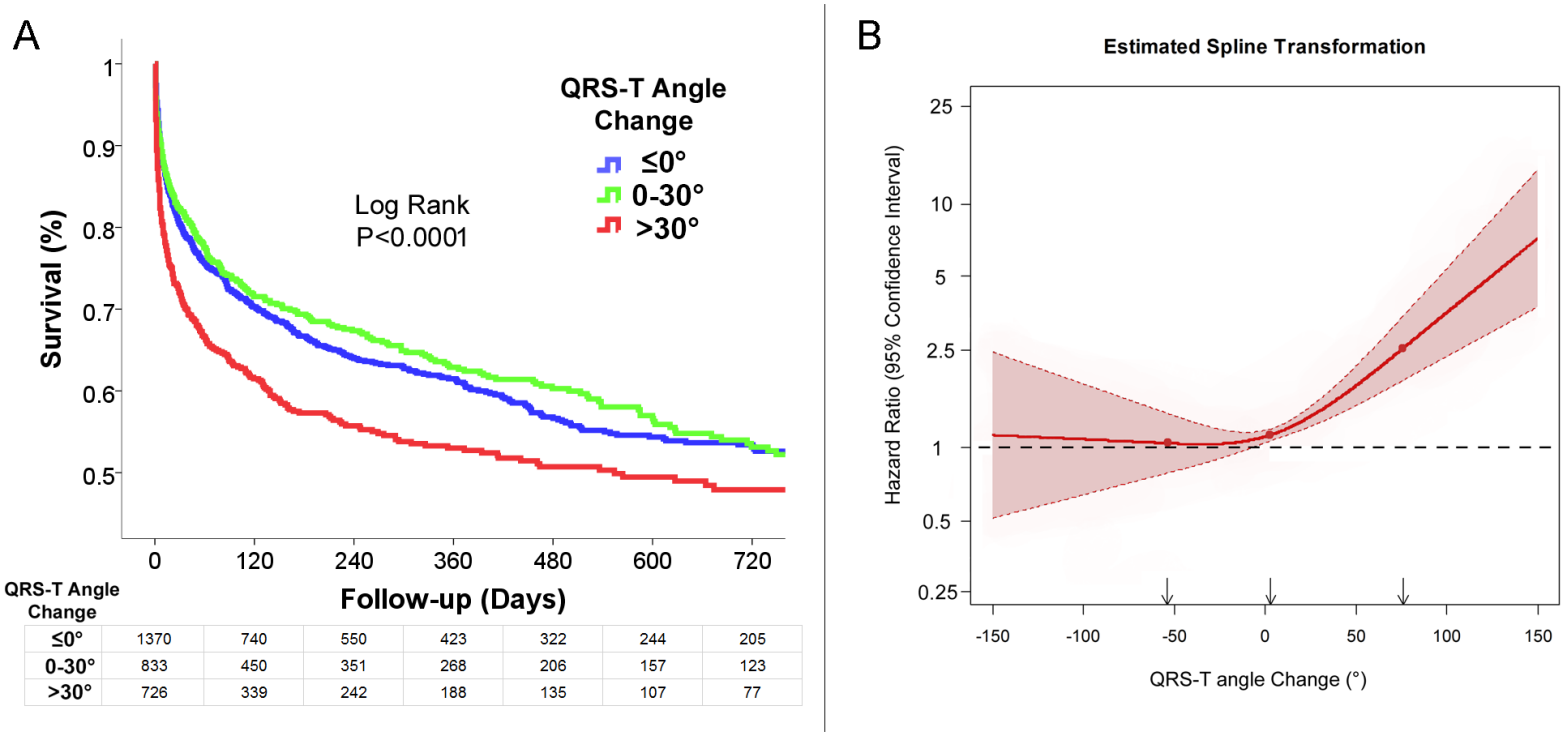
Widening of the QRS-T angle on follow-up was associated with increased mortality. **(A)** Kaplan Meier survival analysis according to QRS-T angle change calculated by the difference between follow-up and baseline ECG. An increase in the QRS-T angle on follow-up above 30° was associated with an increased mortality. The estimated cumulative survival rate at the median follow-up time of 342 days was reduced with an increased QRS-T angle difference; 61.9±1.5% vs 63.6±1.9% vs. 53.3±2.0%, Log rank P<0.00001. **(B)** Cox regression analysis with adjusted hazard ratio for mortality (with 95% confidence interval) of the QRS-T angle change as a continuous variable using restricted cubic splines with 3 knots at the 5^th^, 50^th^ and 95^th^ percentiles of the QRS-T angle change distribution, P<0.0001. Parameters included were parameters outlined in Table 3 with the addition of baseline QRST-T angle.

We analyzed if a change in the QRS-T angle over time had prognostic significance. The median follow-up time from the follow-up ECG was 342 days. A widening in the QRS-T angle i.e. an increase in the QRS-T angle on follow-up was associated with an increase in mortality. The estimated cumulative survival rate at the median follow-up time was reduced with a QRS-T angle increase of more than 30° on follow-up (Fig 3A). Multivariable Cox regression analysis in this cohort after adjustment for significant parameters as detailed in Table 4 demonstrated that an increase in the QRS-T angle was an independent predictor of mortality. A QRS-T angle difference greater than 30° was a significant predictor of mortality (HR 1.52, 95% CI 1.28–1.79, P<0.001; Table 4). Inclusion of HF medications in the multivariable analysis demonstrated again that an increase in the QRS-T angle was a predictor of mortality (HR 1.50, 95% CI 1.27–1.78, P<0.001; Table 4). Analysis, after the exclusion of patients with LBBB or a ventricular-paced rhythm on baseline as well as follow-up ECG (N = 2260), 77% of the cohort, demonstrated a similar result, with a significant increase in mortality with an increase of the QRS-T angle on follow-up (Table 4). A sensitivity analysis including only patients with left ventricular systolic function available (N = 1,306), 45% of the cohort, demonstrated a very similar result (Table 4).

**Table 4:** Hazard ratio for mortality according to the difference in QRS-T angle between follow-up and baseline ECG by Cox regression analysis.

	QRS-T angle difference between follow-up and baseline ECG			
		0°-30°	>30°	P-value
Univariable	1.0 (Reference)	1.48 (0.97–2.27), 0.07	2.22 (1.48–3.33), <0.001	<0.001
Multivariable	1.0 (Reference)	1.02 (0.87–1.20), 0.78	1.52 (1.28–1.79), <0.001	<0.001
Multivariable and Drugs	1.0 (Reference)	1.02 (0.87–1.20), 0.77	1.50 (1.27–1.78), <0.001	<0.001
Analysis after exclusion of patients with LBBB or paced ventricle on baseline or follow-up ECG				
Univariable	1.0 (Reference)	0.89 (0.75–1.05), 0.16	1.42 (1.21–1.66), <0.001	<0.001
Multivariable	1.0 (Reference)	0.97 (0.81–1.15), 0.69	1.54 (1.29–1.84), <0.001	<0.001
Multivariable and Drugs	1.0 (Reference)	0.98 (0.82–1.16), 0.79	1.55 (1.30–1.85), <0.001	<0.001
Sub-analysis of patients with available echocardiographic data regarding left ventricular systolic function				
Univariable	1.0 (Reference)	0.98 (0.80–1.19), 0.81	1.60 (1.32–1.93), <0.001	<0.001
Multivariable	1.0 (Reference)	1.01 (0.81–1.26), 0.96	1.90 (1.52–2.38), <0.001	<0.001
Multivariable and Drugs	1.0 (Reference)	0.98 (0.78–1.22), 0.85	1.87 (1.49–2.34), <0.001	<0.001

Parameters that were included in the models were the parameters outlined in Table 3 with the addition of baseline QRS-T angle.

To further understand the exact impact of the QRS-T angle change on outcome, we performed an analysis that examined the QRS-T angle change in actual degrees as a continuous parameter using restricted cubic splines. Cox regression analysis after adjustment for significant parameters including baseline QRS-T angle demonstrated that an increase in the QRS-T angle was associated with an increase in mortality, evident with an increase of a QRS-T angle difference above 0° (Fig 3B), P<0.0001 for the adjusted model. A very similar result was obtained with the exclusion of patients with LBBB or a ventricular-paced rhythm on either ECG.

### QRS-T angle and left ventricular systolic function

**Fig 4:**
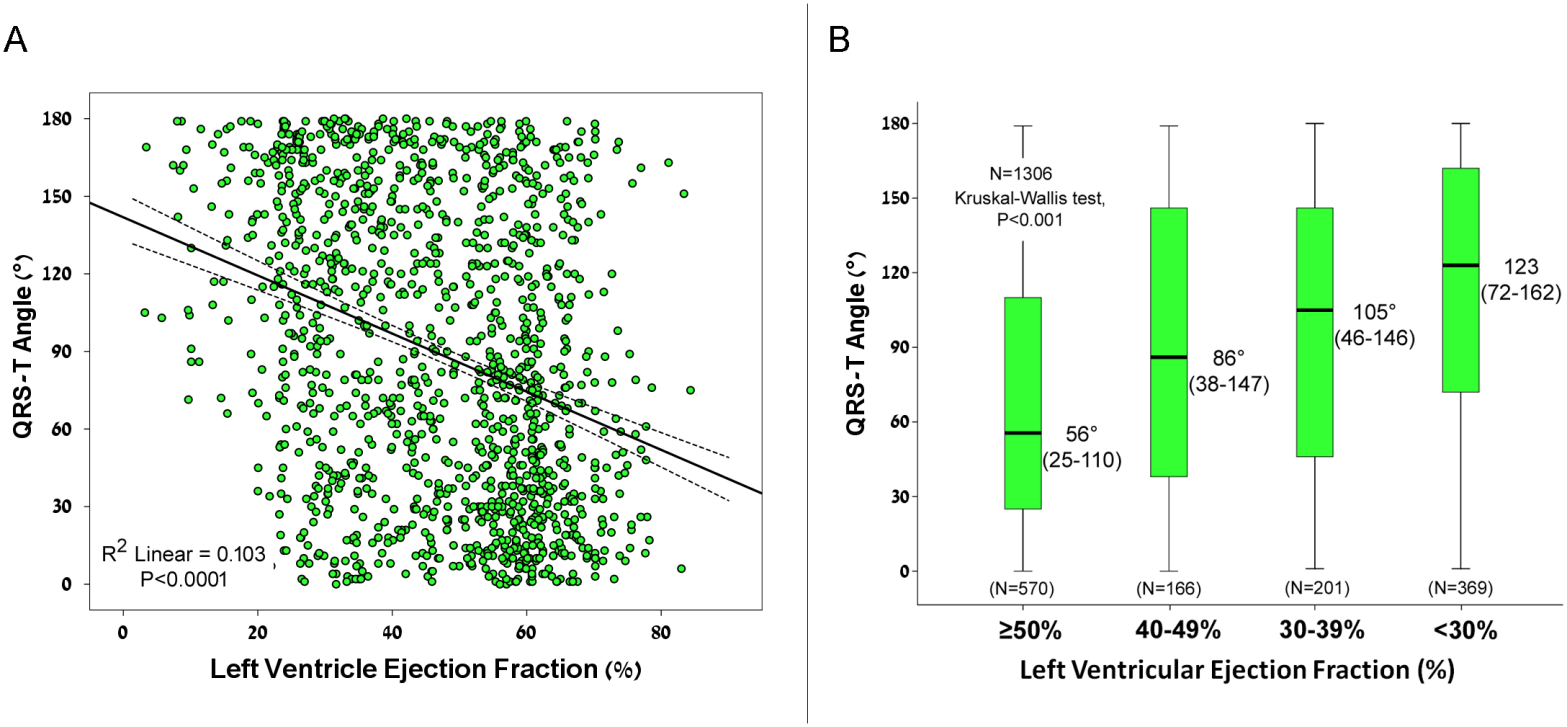
Relation between QRS-T angle and left ventricular systolic function. **(A)** Scatter plot demonstrating an inverse linear relation between QRS-T angle and left ventricular ejection fraction. Linear regression: R^2^ = 0.103, B = -1.06, Standard error 0.11, P<0.0001. **(B)** Box plot of the median QRS-T angle stratified according to the left ventricular ejection fraction. Median QRS-T angle was wider with reduction in left ventricular ejection fraction (Kruskal Wallis test; P<0.001). Box plots denote median and inter-quartile range (IQR); whiskers are of maximum 1.5 IQR.

We analyzed the relation between the baseline QRS-T angle and LV systolic function. The QRS-T angle was associated with a reduction of systolic function. Linear regression analysis demonstrated an inverse association between LVEF and the QRS-T angle (Fig 4A, P<0.0001 for the model). To further demonstrate this relationship, we divided patients into specific LVEF categories and analyzed median QRS-T angle of each category. There was a direct relationship between reduction of left ventricular ejection fraction and a wider QRS-T angle (Kruskal Wallis test, P<0.001; Fig 4B).

## Discussion

The present study on a large cohort of patients with HF, demonstrated that the frontal QRS-T angle was relatively stable in patients with diagnosed HF and has significant prognostic value. The data showed that the frontal QRS-T angle on an initial ECG was a strong predictor of all-cause mortality and is associated with left ventricular systolic function. Also, the present study provides evidence that an increase in the QRS-T angle during follow-up is a predictor of increased mortality and is an ominous sign. Furthermore, there is a direct relation between the magnitude of change in the QRS-T angle during follow-up and mortality.

The novel finding in this study is the temporal changes in the QRS-T angle and its impact on outcome. The present study found that temporal changes in patients with established heart failure are evident but relatively limited. However, an increase in the QRS-T angle over time has prognostic significance and was associated with increased mortality in the present study. The frontal QRS-T angle is an approximation of the spatial QRS-T angle, which is the angle between the spatial axes of ventricular depolarization and repolarization. A greater angle represents a larger discordance between depolarization and repolarization. This signifies abnormal and heterogeneous ventricular repolarization due to damaged or inhomogeneous areas of myocardium, identifying patients with more advanced disease and at a higher risk for ventricular arrhythmias and cardiovascular events. This is consistent with the association between QRS-T angle and left ventricular systolic function seen in the present study. Furthermore, a widening QRS-T angle likely represents a worsening of the underlying pathology and should have an impact on clinical outcome. We did not have detailed information regarding clinical status, ischemic events or left ventricular function during the follow-up, so it is not possible to determine the exact cause of the QRS-T angle change in the present study. Also, it is not possible to say if specific therapy may affect the QRS-T angle. But it is probable that a change with widening of the angle is related to deterioration in the clinical status of the HF patient and this would have prognostic implications as seen in the present study.

Data regarding temporal changes in the QRS-T angle in patients with HF is limited. The only study that analyzed changes was the Defibrillators in Nonischemic Cardiomyopathy Treatment Evaluation (DEFINITE) trial^9^. In that study, 152 patients were evaluated and the QRS-T angle was stable over time as seen in the present study. Widening of the QRS-T angle during follow-up was associated with a decrease in LVEF and an increase in the QRS duration. Conversely, an improvement in NYHA class was associated with narrowing of the QRS-T angle. The above-mentioned study did not evaluate the impact on outcome.

Limitations of the study: The study was an observational study. Follow-up ECG was available in 42% of the cohort and this may introduce bias. The ECG parameters were analyzed using a computer-based algorithm although these algorithms have been clinically validated with a high accuracy. Data on functional capacity and natriuretic peptide levels were not available. Left ventricular ejection fraction was available in only 45% of the cohort.

## Conclusion

The QRS-T angle is relatively stable in patient with HF and is a powerful predictor of outcome. Widening of the QRS-T angle over time is a predictor of mortality. Further prospective research is needed to support the findings of the present study.
